# IL-34 aggravates myocardial ischemia-reperfusion injury by upregulating the HMGB1-IL-17A-IL-6 axis through the JAK signaling pathway

**DOI:** 10.1371/journal.pone.0315489

**Published:** 2025-01-30

**Authors:** Ruisong Ma, Xiaochun Hu, Wenwen Fu, Xiaorong Hu

**Affiliations:** 1 Department of Cardiology, Hainan General Hospital, Haikou, PR China; 2 Hainan Affiliated Hospital of Hainan Medical University, Haikou, PR China; 3 Hainan Clinical Research Center for Cardiology, Haikou, PR China; 4 Department of Cardiology, Renmin Hospital of Wuhan University, Wuhan, PR China; 5 Cardiovascular Research Institute, Wuhan University, Wuhan, PR China; 6 Hubei Key Laboratory of Cardiology, Wuhan, PR China; 7 Department of Cardiology, Zhongnan Hospital of Wuhan University, Wuhan, PR China; Centro Cardiologico Monzino, ITALY

## Abstract

Interleukin-34 (IL-34) was recently reported to be a new biomarker for atherosclerosis diseases, such as coronary artery disease and vascular dementia. IL-34 regulates the expression of proinflammatory cytokines (IL-17A, IL-1 and IL-6), which are classical cytokines involved in myocardial ischemia‒reperfusion (MI/R) injury. However, the exact role of IL-34 in MI/R remains unknown. In this study, a rat MI/R model was used to explore the effect of IL-34 on modulating inflammatory processes during MI/R injury. First, eighteen rats were subjected to 30 min of LAD ligation followed by 0 h, 1 h, 2 h, 4 h, 8 h or 24 h of reperfusion (n = 3 for each group). The level of IL-34 peaked at 4 h after MI/R in the ischemic myocardium. Next, ischemia for 30min and reperfusion for 4h (I/R) model was used. 24 rats were randomly divided into I/R group (n = 8), IL-34+IR group (n = 8) and IL-34+ab12+IR group (n = 8). We found that IL-34 pretreatment increased the expression of inflammatory cytokines, including high mobility group Box 1 (HMGB1), IL-17A, and IL-6; the expression of the apoptosis protein cleaved caspase-3; and the Bcl-2/Bax ratio within the ischemic myocardium. We also observed increased serum cardiac enzymes and a larger myocardial injury area. Treatment with a Janus kinase (JAK) pathway inhibitor, however, partially reduced the expression of these proteins and attenuated myocardial injury. Together, these results showed that IL-34 aggravates MI/R injury by inducing the expression of the HMGB1-IL-17A-IL-6 axis and apoptosis after MI/R, which is partially dependent on the JAK pathway. Therefore, blocking the JAK signaling pathway or inhibiting IL-34 expression might provide a new idea to reduce MI/R injury, but further researches are needed.

## Introduction

Reperfusion therapy is currently the most effective treatment for acute myocardial infarction (AMI). However, the effectiveness of reperfusion therapy might be reduced by 5%~40% by myocardial ischemia‒reperfusion (MI/R) injury [1], for which effective preventive measures are lacking. Thus, a clear understanding of the pathogenesis of MI/R injury is needed. Inflammatory activation, which is initiated during ischemia and aggravated during reperfusion due to the restoration of oxygen-enriched blood flow, participates in pathogenesis and modulates the course of MI/R.

Interleukin-34 (IL-34) is a novel cytokine, first defined in 2008 as the second ligand of colony-stimulating factor-1 receptor (CSF-1R), which has proinflammatory or anti-inflammatory effects by activating the downstream signaling pathway of Janus kinase (JAK), phosphatidylinositol 3-kinase and protein kinase B (PI3K/Akt), nuclear factor kappa B (NF-κB), extracellular-regulated kinase 1/2 (ERK1/2), and STAT, depending on the specific microenvironment [2, 3]. Accumulating studies have revealed increasing serum levels of IL-34 in coronary artery disease (CAD) patients, and IL-34 can predict major adverse cardiovascular events in AMI patients, as well as the prognosis and severity of ischemic cardiomyopathy patients and ischemic stroke patients [4–8]. Moreover, studies have also shown that IL-34 promotes the expression of IL-17A, IL-6 and IL-1β in atherosclerosis, rheumatoid arthritis and inflammatory bowel diseases [9–12]. It has been well established that the high mobility group Box 1 (HMGB1)-interleukin (IL)-17A-IL-6 axis aggravates MI/R injury by promoting the expression of each other and worsening inflammatory waterfalls [13, 14]. The expression of HMGB1 is regulated by the JAK signaling pathway, which is also one of the downstream signals of IL-34-CSF-1R [3]. These studies indicated that IL-34 might be a proinflammatory factor in MI/R injury. The present study was conducted to investigate the effects of IL-34 on MI/R injury and whether the HMGB1-IL-17A-IL-6 axis and JAK signaling pathway are involved in this process.

## Materials and methods

### Rat

Male Sprague–Dawley rats weighing 150–220 g were purchased from Vital River Laboratory (Beijing, China). All rats were raised in the Laboratory Animal Center of Renmin Hospital of Wuhan University with a 12h light/dark cycle, maintained at a temperature of 20–26°C with a relative humidity of 45–65%. The animal experiments in this study complied with the regulations of the National Institutes of Health Guide for the Care and Use of Laboratory Animals and were approved by Renmin Hospital of Wuhan University and Hainan general hospital (No.2021-304).

### Myocardial ischemia/reperfusion (MI/R) model and experimental design

The rat MI/R model was generated as described previously [14]. Briefly, pre-surgical Meloxicam (5 mg/kg intramuscularly, 15 min before the surgery) was administered. The rats were intraperitoneally injected with pentobarbital (30 mg/kg) for anesthesia and to confirm the depth of anesthesia by the lack of the pedal withdrawal reflex. The rats were placed on a warming pad to maintain body temperature. Then, the rats were invasively intubated and ventilated by a volume-controlled rodent ventilator (70 breaths per minute, oxygen concentration 40%, the inspiratory/expiratory ratio was 1:1.5, tidal volume 3–4 mL per 100g rat weight). A left parasternal incision on the chest was made to expose the heart, and the left anterior descending (LAD) coronary artery 3–4 mm from the root was carefully ligated together with a tiny plastic tube lying on the LAD. The chest was temporarily closed with a hemostat. After 30 min of myocardial ischemia, the ligated silk suture was carefully cut off, and the plastic tube was removed to allow myocardial reperfusion for a certain time. Close the thoracic cavity of the rats and suture the skin wound. then put it back into the feeding cage with free water and food.

Step 1: 18 Sprague–Dawley rats were assigned to six groups (n = 3) using a random number table: rats in each group were subjected to 30min of ischemia followed by 0 min, 1h, 2h, 4h, 8h and 24h of reperfusion, respectively.

Step 2: 24 Sprague–Dawley rats were assigned to three treatment groups (n = 8) using a random number table:

Group 1: ischemia and reperfusion group (I/R): rats were subjected to LAD occlusion for 30 min followed by reperfusion for 4 h. After being anesthetized, the rats were treated with phosphate belanced solution (PBS) (1000ul, per rat, i.v., 500ul per femoral vein) 10 minutes before LAD occlusion.

Group 2: IL-34 + I/R group: rats were subjected to LAD occlusion for 30 min followed by reperfusion for 4 h. After being anesthetized, the rats were treated with IL-34 (600ng/kg rat weight, dissolved in PBS, i.v., femoral vein) and PBS (500ul, per rat, i.v., opposite femoral vein) 10 minutes before LAD occlusion.

Group 3: IL-34 + ab12 + I/R group: rats were subjected to LAD occlusion for 30 min followed by reperfusion for 4 h. After being anesthetized, the rats were treated with IL-34 (600ng/kg rat weight, dissolved in PBS, i.v., femoral vein) and ab12 (700 μg/kg rat weight, dissolved in PBS, i.v., opposite femoral vein) 10 minutes before LAD occlusion.

After 4 h of reperfusion, rats were anesthetized with a half dose (15 mg/kg, i.p.) of 2.5% sodium pentobarbital. Next, 2 ml of blood was collected from the jugular vein, the rats were sacrificed by cervical dislocation, and then the heart was excised quickly.

### Reagents and antibodies

The following antibodies were used: recombinant anti-HMGB1 (ab79823, 1:500 dilution), recombinant anti-JAK antibody (ab108596, 1:2000 dilution), GAPDH antibody (ab37168, 1:10000 dilution), cleaved caspase-3 monoclonal antibody (ab214430, 1:2000 dilution), Bcl-2 antibody (ab196495, 1:1000 dilution) and Bax antibody (ab32503, 1:1000 dilution), all of which were purchased from Abcam (Cambridge, UK); and IL-34 polyclonal antibody (bs-18170R, 1:500 dilution), which was purchased from Bioss (Massachusetts, USA). β-actin antibody (TDY051, 1:10000 dilution) was purchased from Beijing TDY Biotech (Beijing, China). HRP-conjugated goat anti-rabbit secondary antibody was purchased from Sungene Biotech (Tianjin, China). Recombinant rat IL-34 protein was purchased from Creative Biomart (Shirley, NY, USA). The JAK inhibitor ab120951 (ab12) was purchased from Abcam (Cambridge, UK). Rat serum creatine kinase MB isoenzyme (CK-Mb) and cTnI isoenzyme assay kits were purchased from Nanjing Jiancheng Bioengineering Institute (Nanjing, China). Rat IL-17A and IL-6 ELISA kits were purchased from Elabscience (Wuhan, China). TUNEL kit was purchased from Merck (Darmstadt, Germany).

### TTC-Ivans blue double staining

After reperfusion for 4h, the LAD was ligated in situ, and 1 ml of 2% Evan blue was injected through the femoral artery. The heart was quickly clipped, rinsed with normal saline, and frozen at -30°C for 30 min. The heart was cut into 5 pieces from the apex to the bottom parallel to the atrioventricular groove, with each piece being 1–2 mm thick. The pieces were placed in a freshly prepared 1% TTC solution and shaken for 15 min in the dark. The infarct area (white) and the risk area (red) were analyzed using an image analyzer (Image-Pro Plus 6.0, Media Cybernetics, Maryland). The infarct size was defined as the percentage of the infarct area/(risk + infarct) area.

### Serum cTnI and CK-MB measurement

After 4h reperfusion, blood (2 ml) was collected from the jugular vein into a BD vacutainer SST (Franklin Lakes, NJ, USA) before the rats were sacrificed. The blood was incubated at room temperature for 30 min to allow clotting, followed by centrifugation at 3000 rpm for 15 min. The supernatant was collected from the rat serum. Rat serum cTnI and CK-Mb levels were assessed using a commercial isoenzyme assay kit according to the manufacturer’s instructions.

### ELISA assay

IL-17A and IL-6 expression levels in myocardial tissue were detected by ELISA using a commercial kit according to the manufacturer’s instructions.

### TUNEL assay

Terminal deoxynucleotidyl transferase dUTP nick end labeling (TUNEL) staining was performed to assess myocardial apoptosis using a commercial kit according to the manufacturer’s instructions. Apoptotic nuclei were dyed brown, and normal nuclei were dyed blue. Five fields at ×400 magnification were randomly selected from each group. The apoptotic index (AI) was defined as (number of brown nuclei per field/total number of nuclei per field) × 100%.

### Western blot analysis of IL-34, HMGB1, cleaved caspase-3, Bcl-2/Bax and JAK protein expression

After 4h reperfusion, venous blood was collected, and the heart was excised quickly. The heart was harvested and flushed with ice-cold PBS until the blood was completely removed. Then, the heart was placed on ice, and the ischemic region on the left ventricular wall was identified and harvested. The tissue was immediately frozen with liquid nitrogen. Western blotting was used to analyze target protein expression as described previously. Briefly, heart tissue was lysed, and total protein was extracted. A bicinchoninic acid kit (Aspen, Wuhan, China) was used for protein determination. Then, the samples were incubated in boiling water for 5 min, separated by 10% SDS‒PAGE and transferred to PVDF membranes. After 1 h of blocking, the membranes were incubated with primary antibodies against rat IL-34, HMGB1, cleaved caspase-3, Bcl-2, Bax, JAK, GAPDH or β-actin at 4°C overnight, followed by incubation with secondary antibodies. Bands with target proteins were scanned and visualized by enhanced chemiluminescence (ECL) (Advansta, USA). Autoradiographs were recorded on X-Omat AR film (Kodak, USA).

### Statistics

GraphPad Prism (San Diego, USA) was used to analyze the statistical values of the data. Before statistical analysis, the F test was used to test equal variance in the data, and Student’s unpaired t test was used to compare two groups; otherwise, an unpaired t test with Welch’s correction was used when the variances were significantly different. For the comparison of multiple groups, one-way ANOVA was used to test the differences among groups, and Dunnett’s multiple comparisons test was used to compare the differences between individual groups and the control group. The data are presented as the mean ± standard deviation (SD). P < 0.05 was considered to indicate statistical significance.

## Results

### Increased expression of IL-34 in MI/R

**Fig 1:**
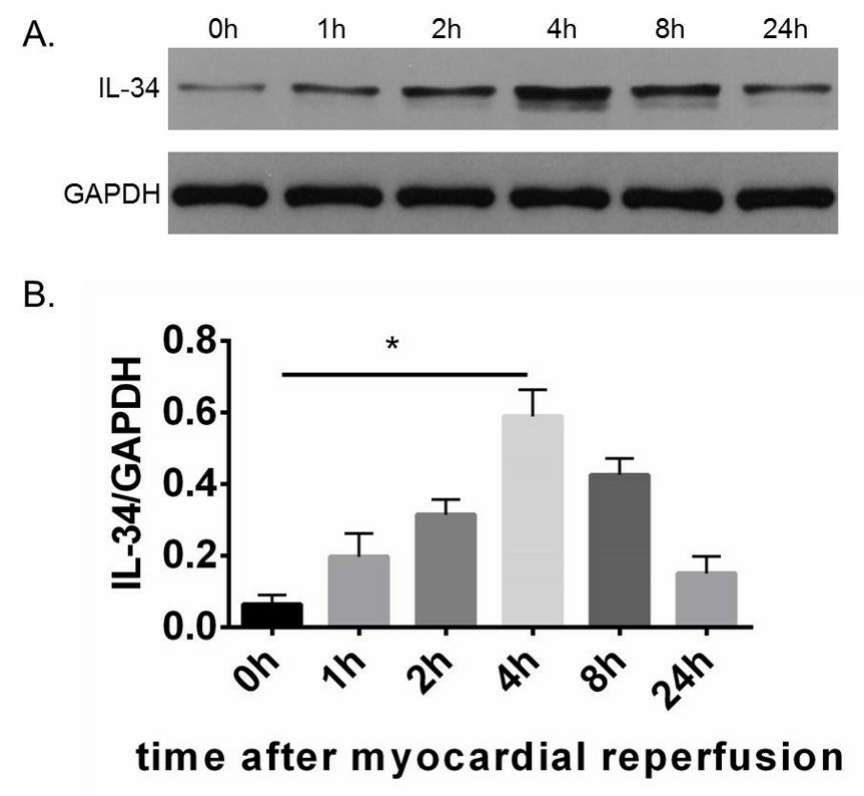
Western blot analysis of IL-34 in myocardial ischemia and reperfusion model. IL-34 expression significantly increases in the myocardium and reaches its peak 4 hours after ischemia and reperfusion. n = 3, *P<0.05.

The relative protein expression of IL-34 in myocardial tissue was measured at 0 h (0.064 ± 0.025), 1 h (0.20 ± 0.065), 2 h (0.31 ± 0.042), 4 h (0.59 ± 0.075), 8 h (0.43 ± 0.046) and 24 h (0.15 ± 0.047) post LAD reperfusion. We found that the IL-34 level gradually increased and peaked at 4 h after MI/R (Fig 1).

### IL-34 aggravates MI/R injury

**Fig 2:**
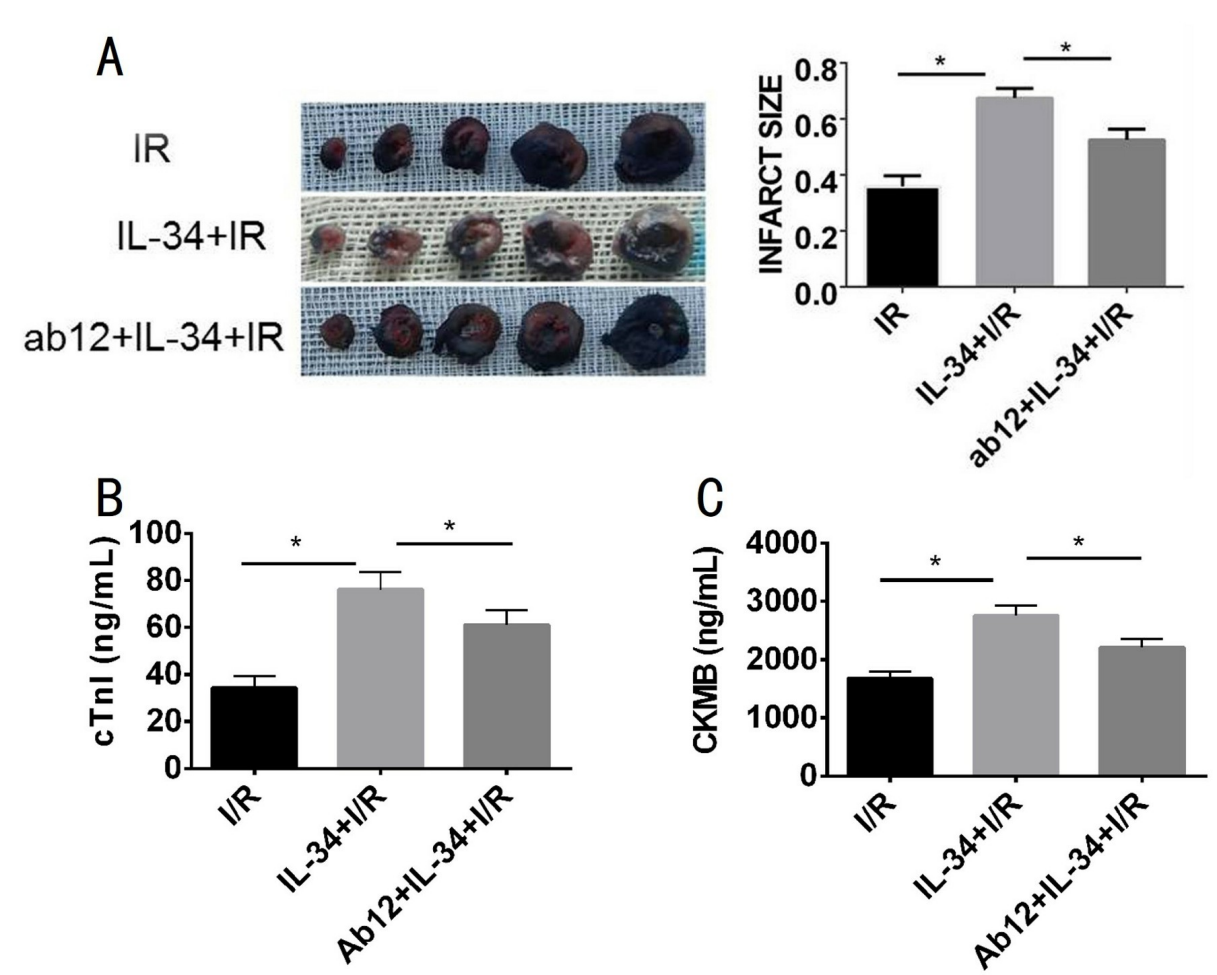
IL-34 aggravates MI/R injury. A: TTC-Ivans blue double staining. IL-34 increses myocardial infarct size after IR, which is partially inhibited by JAK inhibition. The infarct size was defined as the ischemic area/(ischemic area + infarct area) × 100%, n = 3; B and C: Elisa kit assays is used to detecting serum myocardial injury markers. IL-34 increases the expression of cTnI and CK-MB in MI/R, which is partially inhibited by JAK inhibition. MI/R: myocardial ischemia and reperfusion. n = 5.*P<0.05.

Myocardial infarction size, serum cardiac troponin I (cTnI) and CK-MB were measured to evaluate myocardial injury. Compared with those in the IR group, IL-34 treatment significantly increased myocardial infarction size (0.36 ± 0.022 vs 0.67 ± 0.020, p<0.05) and led to increased serum cTnI (76.16 ± 3.26 ng/ml vs 34.12 ± 2.40 ng/ml, p<0.05) and serum CK-Mb (2756.92 ± 78.08 ng/ml vs 1679.13 ± 49.58 ng/mL, p<0.05) levels. However, ab12 partially decreased the IL-34-mediated increase in myocardial infarction size (0.53 ± 0.022, p<0.05), serum cTnI (60.96 ± 2.92 ng/ml, p<0.05) and serum CK-Mb (2215.60 ± 60.75 ng/mL, p<0.05) levels (Fig 2).

### IL-34 aggravates myocardial apoptosis

**Fig 3:**
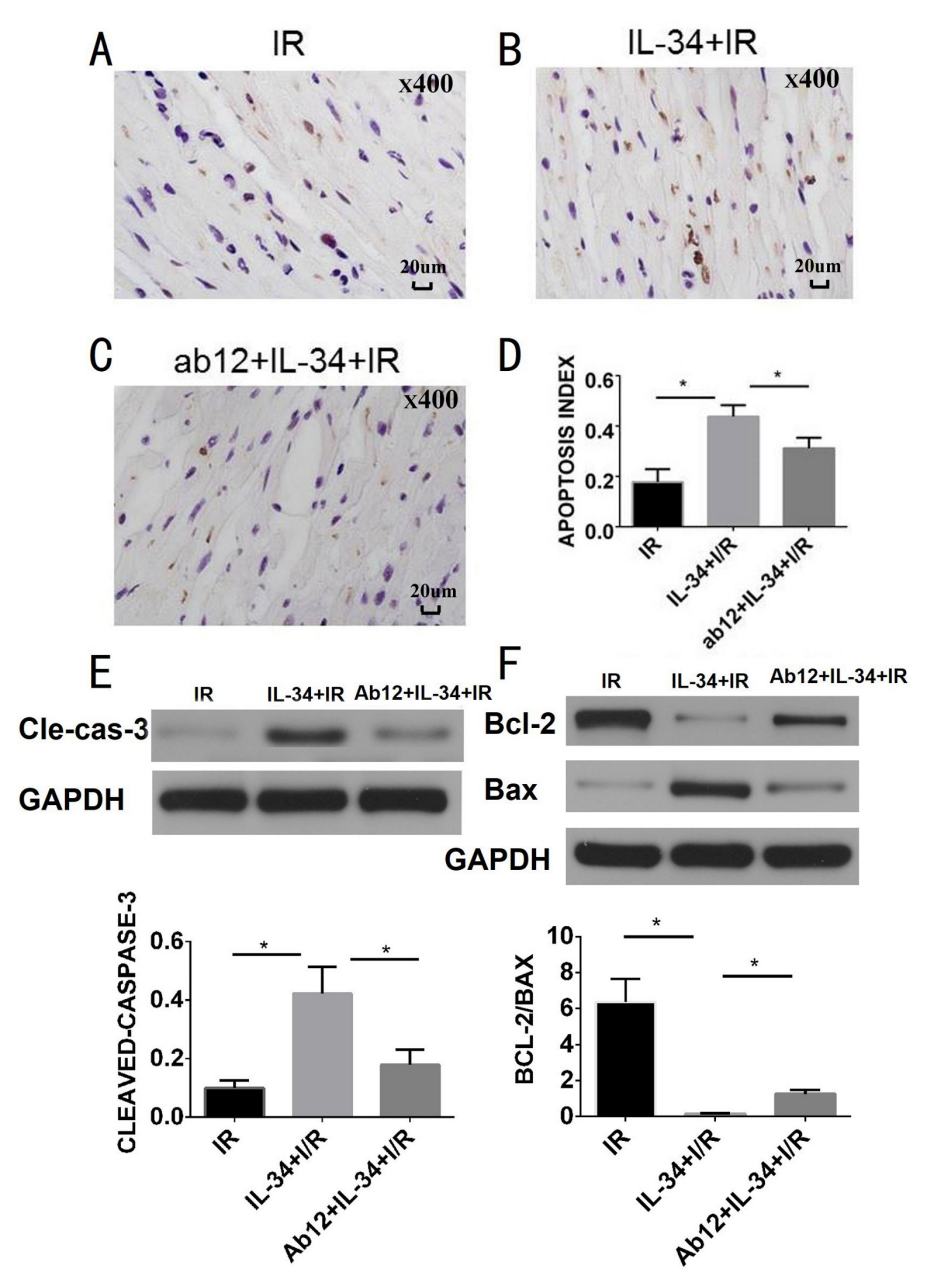
IL-34 aggravates myocardial apoptosis. A, B and C: TUNEL staining in the IR, IL-34+IR and ab12+IL-34+IR groups; D: IL-34 increases myocardial apoptosis after IR, which is partially inhibited by JAK inhibition, n = 5; E and F, western blot is used to detecting apoptosis related protein expression. E: IL-34 increases cleaved caspse-3 expression, which is partially inhibited by JAK inhibition, n = 5; F: IL-34 decreases the ratio of Bcl-2/Bax, which is partially inhibited by JAK inhibition, n = 5. The apoptosis index was defined as (number of brown nuclei per field/total number of nuclei per field) × 100%, *P<0.05.

The AI and the levels of the apoptosis-related proteins cleaved caspase-3, and Bcl-2/Bax were measured to evaluate myocardial apoptosis. Compared with those in the IR group, IL-34 treatment obviously increased the AI (0.44 ± 0.026 vs. 0.18±0.029, p<0.05), cleaved caspase-3 expression (0.44 ± 0.021 vs. 0.071 ± 0.012, p<0.05) and the Bcl-2/Bax ratio (0.14 ± 0.016 vs. 6.36 ± 0.58, p<0.05), which could be partially inhibited by ab12 (AI 0.31±0.024, cleaved caspase-3 0.18 ± 0.019, Bcl-2/Bax 1.25±0.10, all p<0.05) (Fig 3).

### IL-34 activates the JAK signaling pathway and aggravates HMGB1-IL-17A-IL-6 axis expression in MI/R

**Fig 4:**
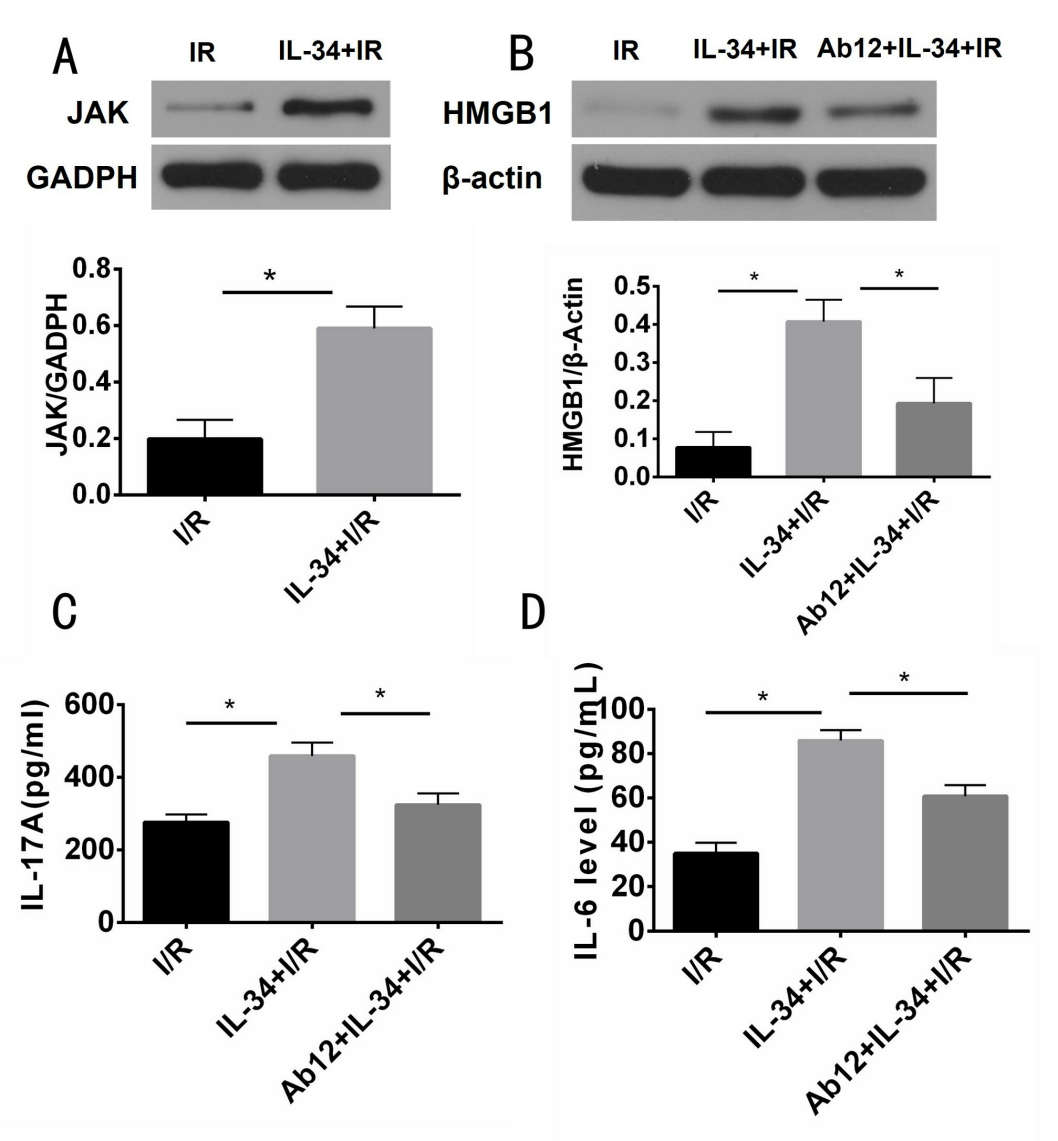
IL-34 regulates the HMGB1-IL-17A-IL-6 axis by activating the JAK signaling pathway; wester bolt assay for HMGB1 and JAK expression, elisa kit assays for IL-17A and IL-6 expression. A: IL-34 significantly increases JAK expression, n = 5; B, C and D: IL-34 significantly increases HMGB1, IL-17A and IL-6 expression, which is partially inhibited by a JAK inhibitor, n = 5. HMGB1: high mobility group protein box-1. *P<0.05.

Compared with those in the IR group, IL-34 treatment markedly increased the expression of JAK (0.59 ± 0.034 vs. 0.20 ± 0.030, p<0.05), HMGB1 (0.41 ± 0.025 vs. 0.077 ± 0.018, p<0.05), IL-17A (458.84± 37.01 pg/ml vs. 276.34 ± 20.91 pg/ml, p<0.05) and IL-6 (85.99 ± 4.68 pg/ml vs. 35.05 ± 4.85 pg/ml, p<0.05). Moreover, ab12 partially inhibited the expression of HMGB1 (0.19 ± 0.030, p<0.05), IL-17A (324.41 ± 31.27, p<0.05) and IL-6 (60.89 ± 4.90 pg/ml, p<0.05) (Fig 4).

## Discussion

### IL-34 is highly expressed in the myocardium in MI/R model

In the steady state, IL-34 transcription occurs in various tissues, including the heart, brain, kidney, spleen, lung, liver, skin and colon. However, the IL-34 protein seems to be expressed in a tissue-specific manner, mainly in the brain, skin and spleen [15–17]. This trend in the expression of the IL-34 protein is altered under pathological conditions, with significantly increased expression of IL-34 at both the mRNA and protein levels in various diseases including coronary artery disease [5], type 2 diabetes [18], rheumatoid arthritis [19, 20], systemic lupus erythematosus [21] and inflammation [2]. In the pathological state, IL-34 is secreted by fibroblasts, macrophages, and the endothelium under the stimulation of chemical stressors, pro-inflammatory cytokines or DNA damaging agents [22]. Accumulating studies have shown that the IL-34 concentration is significantly increased in patients with CAD, and this increase is positively correlated with the severity of acute myocardial infarction [5, 6]. At the cellular level, fibroblast, macrophage and the endothelium might be sources of IL-34. The present study further demonstrated that the IL-34 protein level in myocardial tissue was increased in the MI/R model and peaked level at 4 h after reperfusion, which suggests that IL-34 is abundantly expressed in the MI/R model. So, the MI/R model (ischemia for 30min and reperfusion for 4h) was selected in the present study.

### IL-34 aggravates MI/R injury by promoting myocardial inflammation and myocardial apoptosis

IL-34 is a multifunctional cytokine, that exerts pro- or anti-inflammatory effects depending on the specific microenvironment [2, 3]. Zwicker and Xu reported that IL-34 might inhibit TNF-α expression in macrophages 1 and overlap with the anti-inflammatory cytokine IL-10-secreting macrophages in inflammatory bowel disease and infectious disease models, suggesting the anti-inflammatory potential of IL-34 [23, 24]. However, the majority of studies have shown that IL-34 could be a pro-inflammatory factor in kidney injury, autoimmune diseases, cancers, infectious disease and MI/R [2, 25, 26]. IL-34 could promote atherogenesis by enhancing foam cell formation and IL-6 expression [11]. Huang et al. [8] demonstrated that IL-34 contributed to neuroinflammation and might predict the severity and prognosis of ischemic stroke. Qin et al. [27] suggested that IL-34 directly and indirectly aggravates inflammatory responses, interstitial fibrosis and cardiac function during the post-MI period. Xin et al suggested that IL-34 could upregulate the expression of IL-17A, IL-6 and TNF-α by regulating IL-17 in fibroblast-like synoviocytes, which further affects their proliferation, apoptosis and function [19]. The present study revealed that IL-34 could upregulate IL-17A and IL-6 expression in myocardial tissue, which is consistent with previous studies. In addition, we first propose that IL-34 might upregulate HMGB1 expression. Thus, we hypothesize that IL-34 could upregulate the HMGB1-IL-17A-IL-6 axis, suggesting that it could be a pro-inflammatory cytokine in MI/R.

The HMGB1-IL-17A-IL-6 axis aggravates MI/R injury directly via the induction of inflammatory waterfalls or indirectly via the induction of apoptosis, myocardial autophagy, and oxidative stress [13, 14]. Once released from the cell, HMGB1 binds to its receptor, RAGE, and regulates IL-17A and IL-6 expression via the PI3K/Akt, MAPK, and NF-kappaB signaling pathways. IL-17A and IL-6 could in turn aggravate HMGB1 release. This inflammatory cascade further induces cardiomyocyte apoptosis and injury [14]. The present study revealed that IL-34 significantly increased HMGB1-IL-17A-IL-6 axis expression and induced cardiomyocyte apoptosis. Thus, IL-34 might aggravate MI/R injury by upregulating the HMGB1-IL-17A-IL-6 axis.

### IL-34 regulates the HMGB1-IL-17A-IL-6 axis by activating the JAK signaling pathway

The main receptor for IL-34 is macrophage colony-stimulating factor receptor (CSF-1R). IL-34/CSF-1R participates in the proliferation, migration and cytokine expression of macrophages and fibroblasts by activating downstream signaling pathways, including the JAK [2, 28, 29]. HMGB1, an early proinflammatory protein, can be actively secreted under stimulation by Lipopolysaccharide (LPS), TNF-α, infection and endogenous host stimuli or passively released under conditions of cell apoptosis and necrocytosis [30]. LPS and TNF-α induce the active secretion of HMGB1 via the JAK signaling pathway in a time-dependent manner [30], which is also the downstream signaling pathway of IL-34/CSF-1R. The main sources of HMGB1 in the heart are M1 macrophages and myocardial fibroblasts. IL-34 can induce proinflammatory M1 macrophage differentiation when incubated with LPS [2]. IL-34 also exacerbates MI/R injury by facilitating macrophage recruitment and polarization [26]. In addition, Xin et al suggested that IL-34 could upregulate the expression of IL-17A, IL-6 and TNF-α by regulating IL-17 in fibroblast-like synoviocytes, further affecting their proliferation, apoptosis and function [19]. IL-34 increased the expression of two cytokines, IL-17A and IL-6, in HMBG1-IL-17A-IL-6 axis and upregulated the signaling pathway upstream of HMGB1. For the first time, the present study proposed that IL-34 could promote HMGB1 release, which could be partly inhibited by a JAK signaling pathway inhibitor. Thus, we suggest that IL-34 might promote HMGB1 release via the JAK signaling pathway.

In conclusion, the present study demonstrated that 1) IL-34, which is highly expressed in MI/R, could aggravate MI/R injury by upregulating the HMGB1-IL-17A-IL-6 axis. 2) IL-34 might upregulate the HMGB1-IL-17A-IL-6 axis by activating the JAK pathway. Therefore, IL-34 might not only be a biomarker for poor prognosis in patients with coronary heart disease, but also a target for reducing MI/R injury. However, further researches are needed.

### Limitations

The present study has two main limitations. First, the small sample size (n = 3 or n = 5) in biological studies may develop a risk of bias, and further studies and large sample sizes are needed. Second, research is focused on one time point of four hours after reperfusion and one type of animal. The effects of IL-34 on MI/R injury should be investigated and verified on different time point of ischemia and reperfusion, and different type of animals.

## Supporting information

S1 Raw image (PDF)

S1 Raw data (PDF)
