# Supplementary figures and images for: IL-34 aggravates myocardial ischemia-reperfusion injury by upregulating the HMGB1-IL-17A-IL-6 axis through the JAK signaling pathway
Source: PLoS One. 2025 Jan 30;20(1):e0315489. doi: 10.1371/journal.pone.0315489 (PMC11781702; doi:10.1371/journal.pone.0315489)

IL-34 for Fig 1.

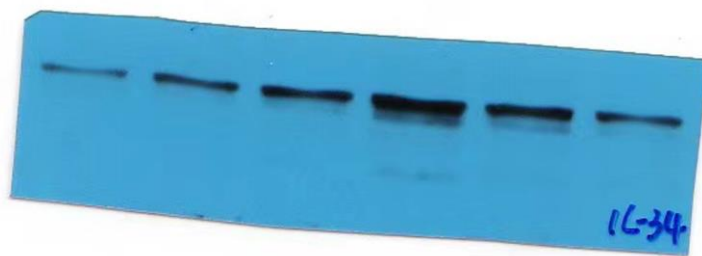

Cleaved-caspase3 for Fig 3E

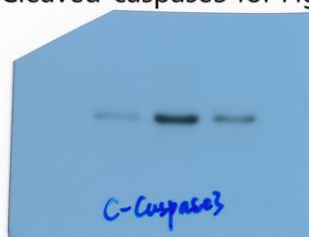

BCL-2 and Bax for Fig 3F

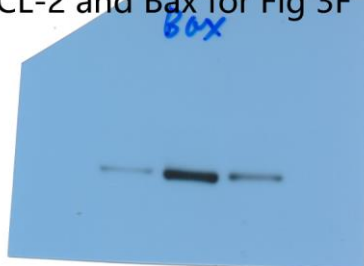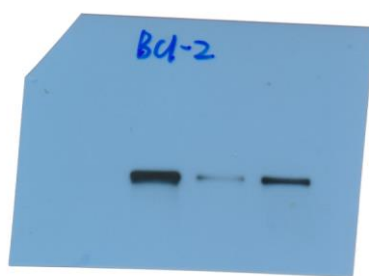

HMGB1 for Fig 4B

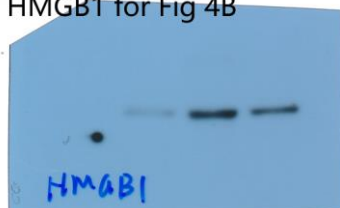

Supplement: S1 Raw image — (PDF) [file pone.0315489.s001.pdf]
