# Supplementary material for: IL-34 aggravates myocardial ischemia-reperfusion injury by upregulating the HMGB1-IL-17A-IL-6 axis through the JAK signaling pathway
Source: PLoS One. 2025 Jan 30;20(1):e0315489. doi: 10.1371/journal.pone.0315489 (PMC11781702; doi:10.1371/journal.pone.0315489)
Supplement: S1 Raw data — (PDF) [file pone.0315489.s002.pdf]

IL-34 expression after IR

0h	1h	2h	4h	8h	24h
0.093	0.14	0.311	0.509	0.408	0.165
0.048	0.201	0.268	0.625	0.478	0.098
0.051	0.269	0.351	0.649	0.392	0.188

IL-34 aggravates MIR injury

Infarct Size	IR	IL-34+IR	ab12+IL-34+IR	cTnI	IR	IL-34+IR	ab12+IL-34+IR
	0.35	0.668	0.522		33.8	79.78	57.85
	0.384	0.697	0.507		31.12	75.67	60.74
	0.342	0.659	0.551		33.02	78.15	65.51
					37.56	76.09	61.57
					35.12	71.14	59.14

CK-MB	IR	IL-34+IR	ab12+IL-34+IR
	1643.23	2801.12	2235.51
	1735.63	2845.32	2305.18
	1634.58	2788.27	2187.47
	1651.87	2664.95	2208.38
	1730.34	2684.96	2141.44

IL-34 aggravates myocardium apoptosis

apoptosis i	IR	IL-34+IR	ab12+IL-34+IR	cleaved-ca	IR	IL-34+IR	ab12+IL-34+IR
	0.146	0.456	0.278		0.058	0.456	0.183
	0.178	0.407	0.335		0.079	0.467	0.161
	0.224	0.471	0.313		0.057	0.426	0.154
	0.165	0.422	0.287		0.075	0.417	0.207
	0.186	0.441	0.322		0.084	0.444	0.181

Bcl-2/Bax	IR	IL-34+IR	ab12+IL-34+IR
	6.764	0.13	1.208
	6.958	0.166	1.099
	5.471	0.125	1.278
	6.183	0.13	1.322
	6.407	0.146	1.347

IL-34 activates JAK signaling pathway and aggravates HMGB1-IL-17A-IL-6 axis expression in MI/R

JAK	IR	IL-34+IR
	0.187	0.559
	0.194	0.566
	0.185	0.597
	0.158	0.576
	0.24	0.643

HMGB1	IR	IL-34+IR	ab12+IL-34+IR
	0.075	0.427	0.176
	0.098	0.433	0.232
	0.087	0.414	0.159
	0.051	0.371	0.185
	0.074	0.396	0.218

IL-17A	IR	IL-34+IR	ab12+IL-34+IR	IL-6	IR	IL-34+IR	ab12+IL-34+IR
	283.26	408.24	344.37		36.14	89.85	64.67

270.95	491.16	330.52		42.95	83.82	61.36
308.28	493.93	361.54		33.34	91.27	54.82
266.37	466.28	299.96		30.68	79.68	66.44
252.84	434.58	285.68		32.12	85.31	57.17
